# 

**Published:** 1866-08

**Authors:** 


### ORIGINAL CONTRIBUTIONS.

ART. XXV. On the Function of the Minute Arteries. By J. S. Jewell, M.D., Prof. of Anatomy in Chicago Medical College, ....... 449

ART. XXVI. A New Plastic Operation for Certain Deformities of the Face. By E. Andrews, M.D., Professor of Surgery in Chicago Medical College, ....... 465

ART. XXVII. Remarks on Dr. Wright's Theory of Cholera. By D. W. Flora, M.D., of Chicago, ....... 470

ART. XXVIII. Report on the Prevalence of Diseases, in the City of Chicago, from April 1st, to June 30th, 1866. By N. S. Davis, M.D., Committee, ....... 475

ART. XXIX. Two Cases of Angular Curvature, Supervening upon Lateral Curvature of the Spine. By F. O. Earle, M.D., of Chicago, ....... 479

### PROCEEDINGS OF SOCIETIES.

Medical Convention, ....... 481

Annual Meeting of Rock River Union Medical Society, ....... 484

Clinton Medical Society, ....... 486

### SELECTIONS.

On a New and Ready Mode of Producing Local Anæsthesia. By Benj. W. Richardson, M.A., M.D., F.R.C.P., Senior Physician to the Royal Infirmary for Diseases of the Chest, ....... 487

Remarks Concerning some of the Diseases Prevailing among the Freed-people in the District of Columbia. (Bureau Refugees, Freedmen, Abandoned Lands.) By Robert Reyburn, Surgeon, U.S. Vols., ....... 494

### BOOK NOTICES.

A Manual of the Principles of Surgery, Based on Pathology, for Students. By William Canniff, Licentiate of the Medical Board of Upper Canada, 498

Reflex Paralysis: Its Pathological Anatomy and Relations to the Sympathetic Nervous System. By M. Gonzales Echeverria, M.D., ....... 499

Recent Advances in Ophthalmic Science. The Boylston Prize Essay for 1865. By Henry W. Williams, M.D., Ophthalmic Surgeon to the City Hospital, Boston, ....... 499

Asiatic Cholera: Its Origin and Spread in Asia, Africa, and Europe; Introduction into America through Canada; Remote and Proximate Causes, Symptoms, and Pathology, and the Various Modes of Treatment Analysed. By R. Nelson, M.D., Health Commissioner during the first two invasions, 1832-4, ....... 499

Asiatic Cholera. By F. A. Burral, M.D., ....... 499

Diarrhœa and Cholera: Their Origin, Proximate Cause and Cure, through the Agency of the Nervous System, by Means of Ice. By John Chapman, M.D., M.R.C.P., M.R.C.S. ....... 500

### EDITORIAL.

Chicago Medical College, ....... 500

Proceedings of the Illinois State Medical Society, ....... 502

Chicago Medical Society, ....... 502

European Medical News, ....... 503

Sulphites in Malarial Fevers. Letter from W. H. Baxter, M.D., Moscow, 503

Physicians Report of St. Luke's Hospital, from October 18th, 1865, to March 31st, 1866, ....... 504

Mortality of the City of Chicago for June, 1866, ....... 505

Biographical Dictionary, ....... 506

Prevention of Cholera, ....... 506

Stevens' Triennial Prize, ....... 507

Annual Prizes of the Imperial Academy of Medicine, ....... 508

Obituary Record, ....... 508

Died, at Jaffa, Dr. Thomas Hodgkin, ....... 509

